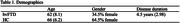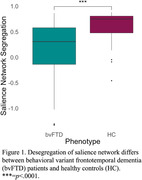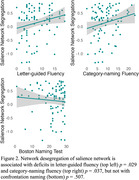# Structural desegregation of salience network is associated with executive functioning deficits in behavioral variant frontotemporal dementia

**DOI:** 10.1002/alz.091801

**Published:** 2025-01-09

**Authors:** Melanie A Matyi, Hamsi Radhakrishnan, Jeffrey S Phillips, Philip A. Cook, Emma Rhodes, James C. Gee, David J Irwin, Corey T McMillan, Lauren Massimo

**Affiliations:** ^1^ University of Pennsylvania, Philadelphia, PA USA; ^2^ Penn Frontotemporal Degeneration Center, Department of Neurology, Perelman School of Medicine, University of Pennsylvania, Philadelphia, PA USA; ^3^ University of Pennsylvania, School of Nursing, Philadelphia, PA USA

## Abstract

**Background:**

The human brain is organized in dense and distinct intrinsic networks that are topographically arranged and mediate particular cognitive functions. The characteristic of intrinsic network organization that supports this functional specialization of cognitive domains is known as modular segregation. Neurodegeneration is associated with changes in brain network organization that contribute to cognitive decline among healthy older adults but network segregation has rarely been studied in behavioral variant frontotemporal dementia (bvFTD). Examining segregation may provide insights into network organization and its contribution to cognition in bvFTD. We hypothesize that desegregation of structural networks will differ between bvFTD and healthy controls (HC) and will be associated with deficits in high‐executive demand cognitive tasks in bvFTD patients.

**Methods:**

86 bvFTD and 37 HC (see Table 1) underwent diffusion MRI. Patients also completed tests of letter‐guided fluency, category‐naming fluency, and the Boston Naming Test (BNT). Structural connectivity was derived from deterministic tracking among 100 cortical regions mapped to 7 intrinsic networks, defined by the Schaefer atlas, using the DSI‐Studio generalized q‐sampling imaging pipeline as implemented in QSIPrep. Network segregation was characterized by high within‐network connectivity but low between‐network connectivity. To identify patterns of desegregation in structural networks, one‐way ANOVAs were conducted between bvFTD and HC groups for each network, controlling for age and motion. Linear regression was then performed to assess whether desegregation was associated with executive performance, controlling for disease duration and education.

**Results:**

Desegregation of the salience network was observed in bvFTD, relative to HC (see Figure 1) and was associated with deficits in tasks with high executive demand (letter‐guided and category‐naming fluency), but not with performance on the BNT, a task with lower executive demand (see Figure 2).

**Conclusions:**

Results indicate that structural desegregation of salience network is characteristic of bvFTD, reflecting a reduced capacity for specialized processing. Salience network desegregation was also uniquely associated with performance on cognitive tasks requiring high demand on executive resources. Overall, findings underscore the importance of salience network in bvFTD pathology. Critically, results demonstrate the executive consequences of network desegregation.